# Correction: Temporal Gillespie algorithm: Fast simulation of contagion processes on time-varying networks

**DOI:** 10.1371/journal.pcbi.1007190

**Published:** 2019-07-03

**Authors:** Christian L. Vestergaard, Mathieu Génois

There is an error in Pseudocode 1, and 2, where a line was omitted after line 46 (in 1), 61 (in 2). The following line was omitted:

tau - = xi*Lambda //subtract remainder of time-step

**Pseudocode 1:**
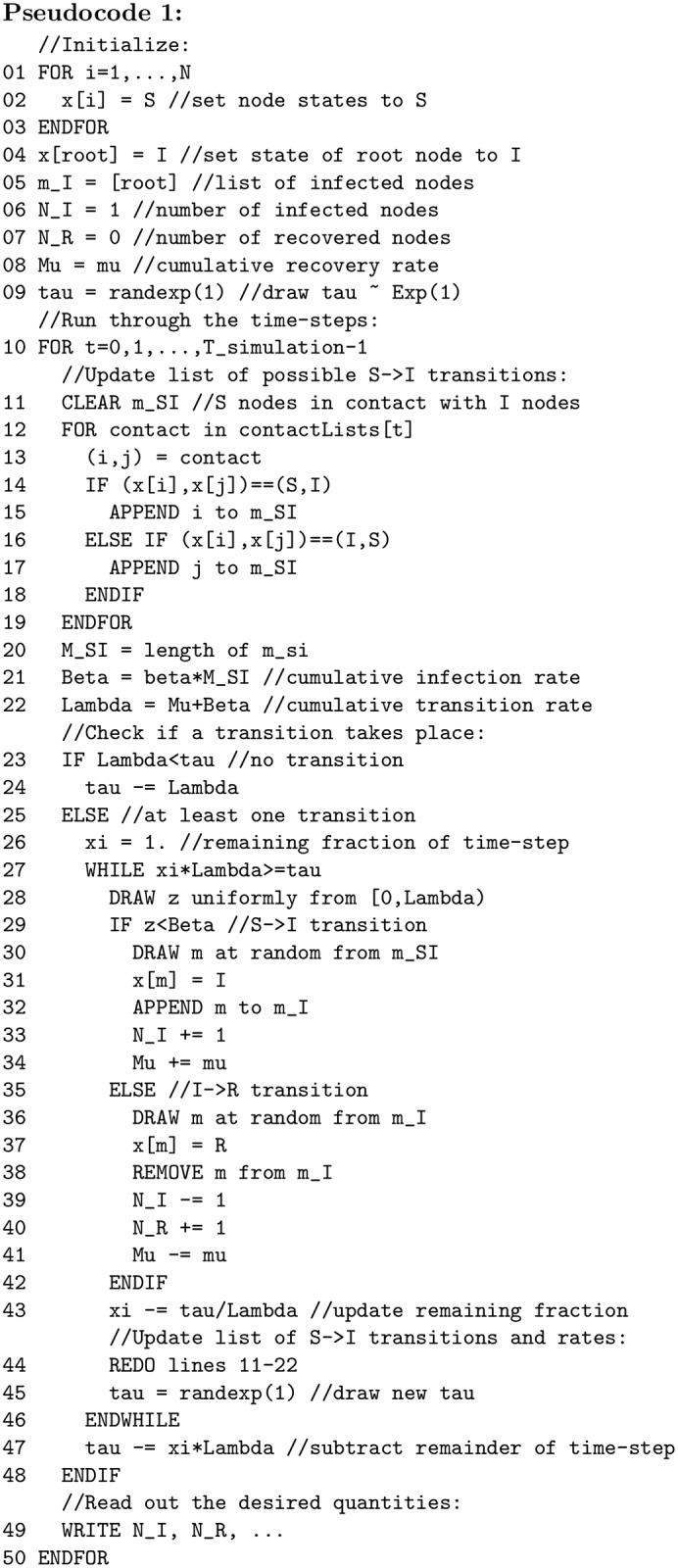
Pseudocode for an SIR process with constant and homogeneous transition rates. C++ code for homogeneous and heterogeneous populations is given in S1 Files.

**Pseudocode 2:**
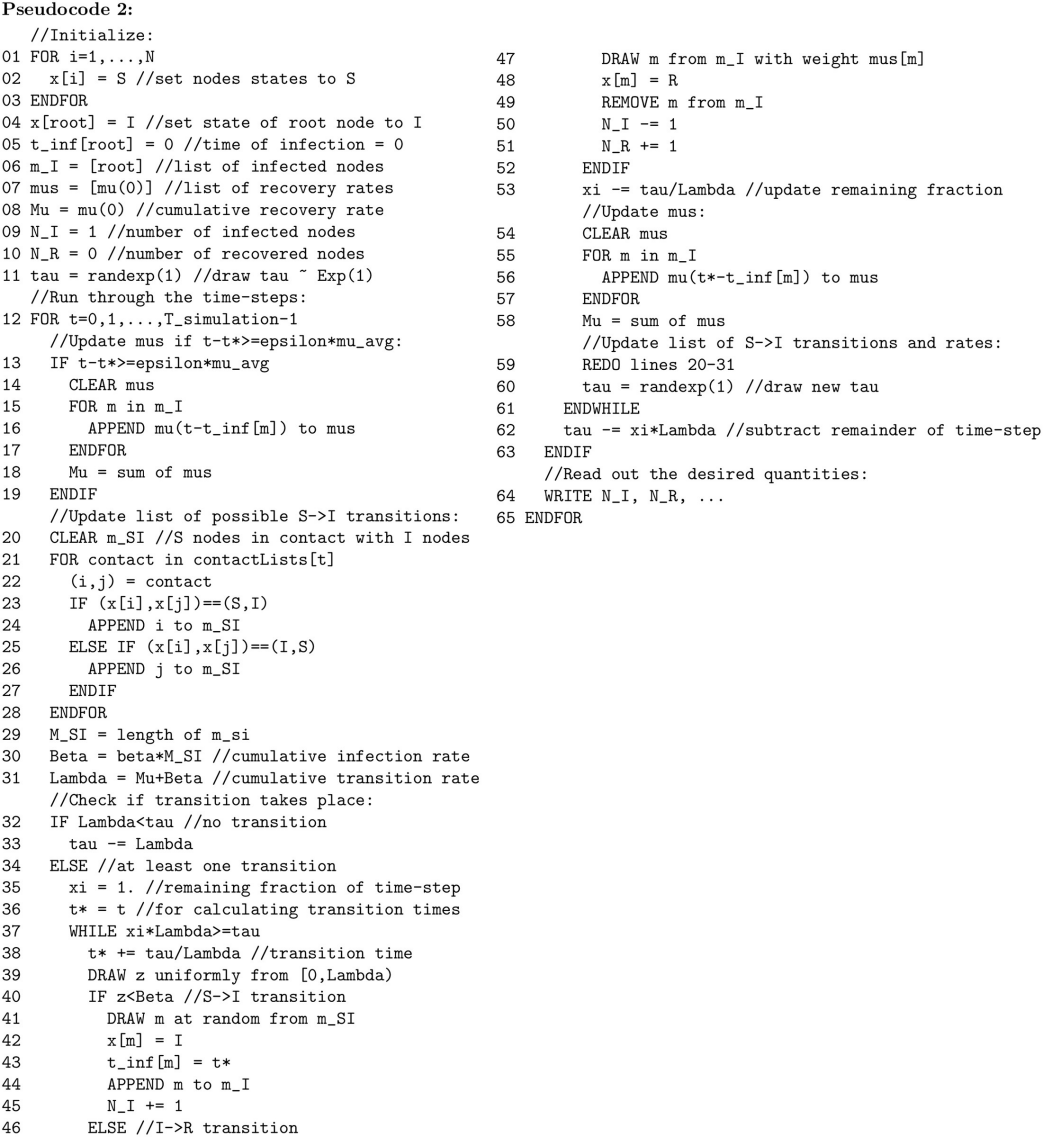
Pseudocode for a non-Markovian SIR process with non-constant recovery rates. The function mu returns the instantaneous recovery rate as function of (t–t*); for Weibull distributed recovery times, mu is given by Eq. (23). C++ code is given in S1 Files.

This change in the pseudocode results in changes to supporting information S1, S2 and S3 Figs. The corrected supporting information figures are:

## Supporting information

S1 Fig Numerical results from temporal Gillespie and rejection sampling algorithms for contagion processes taking place on empirical networks. (A)–(D) for a SIR process and (E)–(H) a SIS process. (A),(B),(E), and (F) for *β*Δ*t* = 10^−2^ and *μ*Δ*t* = 10^−4^; (C),(D),(G), and (H) for *β*Δ*t* = 10^−1^ and *μ*Δ*t* = 10^−3^. (A),(C) Mean number of nodes in each state of the SIR model as function of time. (B),(D) Distribution of final epidemic size (number of recovered nodes when *I* = 0) in the SIR model. (E),(G) Mean number of nodes in each state of the SIS model as function of time. (F),(H) Distribution of the number of infected nodes in the stationary state (*t* →∞) of the SIS model. All simulations were performed 1 000 000 times with the root node chosen at random on a face-to-face contact network recorded in a high school (Table 1).
(PDF)

S2 Fig Comparison of numerical results from temporal Gillespie and rejection sampling algorithms for high transition probability per time-step. (A)–(D) for a SIR process and (E)–(H) a SIS process. (A),(B),(E), and (F) for *β*Δ*t* = 10^−1^ and *μ*Δ*t* = 10^−3^; (C),(D),(G), and (H) for *β*Δ*t* = 1 and *μ*Δ*t* = 10^−2^. (A),(C) Mean number of nodes in each state of the SIR model as function of time. (B),(D) Distribution of final epidemic size (number of recovered nodes when *I* = 0) in the SIR model. (E),(G) Mean number of nodes in each state of the SIS model as function of time. (F),(H) Distribution of the number of infected nodes in the stationary state (*t* →∞) of the SIS model. All simulations were performed 1 000 000 times with the root node chosen at random on an activity driven network consisting of *N* = 100 nodes, with activities *a*_*i*_ = *ηz*_*i*_, where *η* = 0.1 and z_i_ ~ z_i_^-3.2^ for z_i_ ϵ [0.03,1), and a node formed two contacts when active.
(PDF)

S3 Fig Comparison of numerical results from temporal Gillespie and rejection sampling algorithms for a non-Markovian SIR process. (A),(C) Mean number of nodes in each state as function of time in the SIR model with Weibull distributed recovery times (Sec. 6: “Non-Markovian processes”); the parameter controlling the precision of the temporal Gillespie algorithm was set to *ϵ* = 0 (quasi-exact). (B),(D) Distribution of final epidemic size (number of recovered nodes when *I* = 0). (A),(B) *βΔt* = 10^−2^ and *μΔt* = 10^−4^; (C),(D) *βΔt* = 10^−1^ and *μΔt* = 10^−3^. The outcome of the rejection sampling algorithm approaches that of the temporal Gillespie algorithm as *βΔt* and *μΔt* become smaller. All simulations were performed 100 000 times with the root node chosen at random on an activity driven network consisting of *N* = 100 nodes, with activities *a*_*i*_ = *ηz*_*i*_, where *η* = 0.1 and z_i_ ~ z_i_^-3.2^ for z_i_ ϵ [0.03,1), and a node formed two contacts when active. Nodes’ recovery times followed Eq. (20) with *γ* = 1.5 and the length of a time-step was *Δt* = 1 s.
(PDF)
