# Supplementary figures and images for: Correction: Temporal Gillespie algorithm: Fast simulation of contagion processes on time-varying networks
Source: PLoS Comput Biol. 2019 Jul 3;15(7):e1007190. doi: 10.1371/journal.pcbi.1007190 (PMC6608897; doi:10.1371/journal.pcbi.1007190)

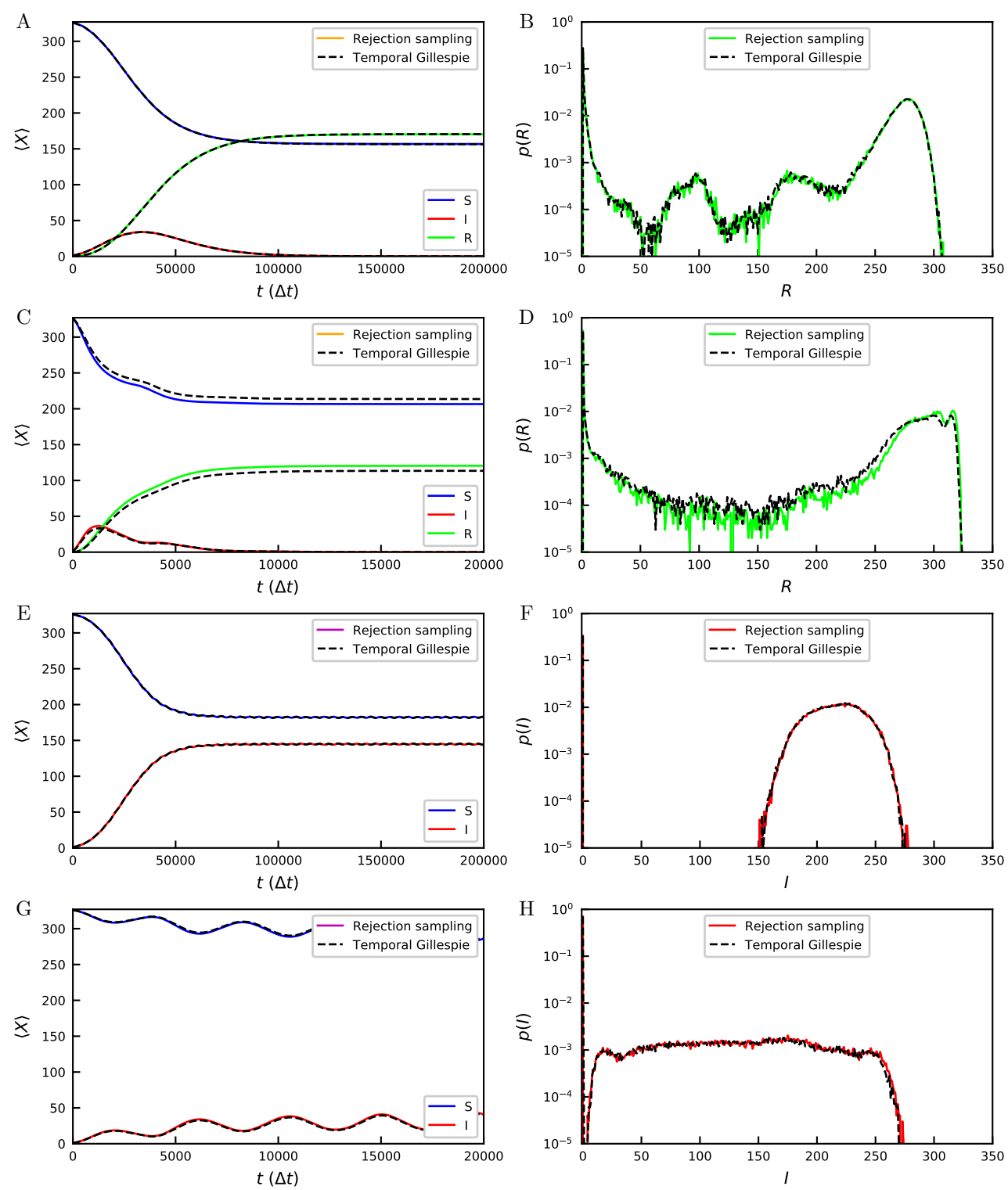

Supplement: S1 Fig — (A)–(D) for a SIR process and (E)–(H) a SIS process. (A),(B),(E), and (F) for βΔt = 10−2 and μΔt = 10−4; (C),(D),(G), and (H) for βΔt = 10−1 and μΔt = 10−3. (A),(C) Mean number of nodes in each state of the SIR model as function of time. (B),(D) Distribution of final epidemic size (number of recovered nodes when I = 0) in the SIR model. (E),(G) Mean number of nodes in each state of the SIS model as function of time. (F),(H) Distribution of the number of infected nodes in the stationary state (t →∞) of the SIS model. All simulations were performed 1 000 000 times with the root node chosen at random on a face-to-face contact network recorded in a high school (Table 1). (PDF) [file pcbi.1007190.s001.pdf]

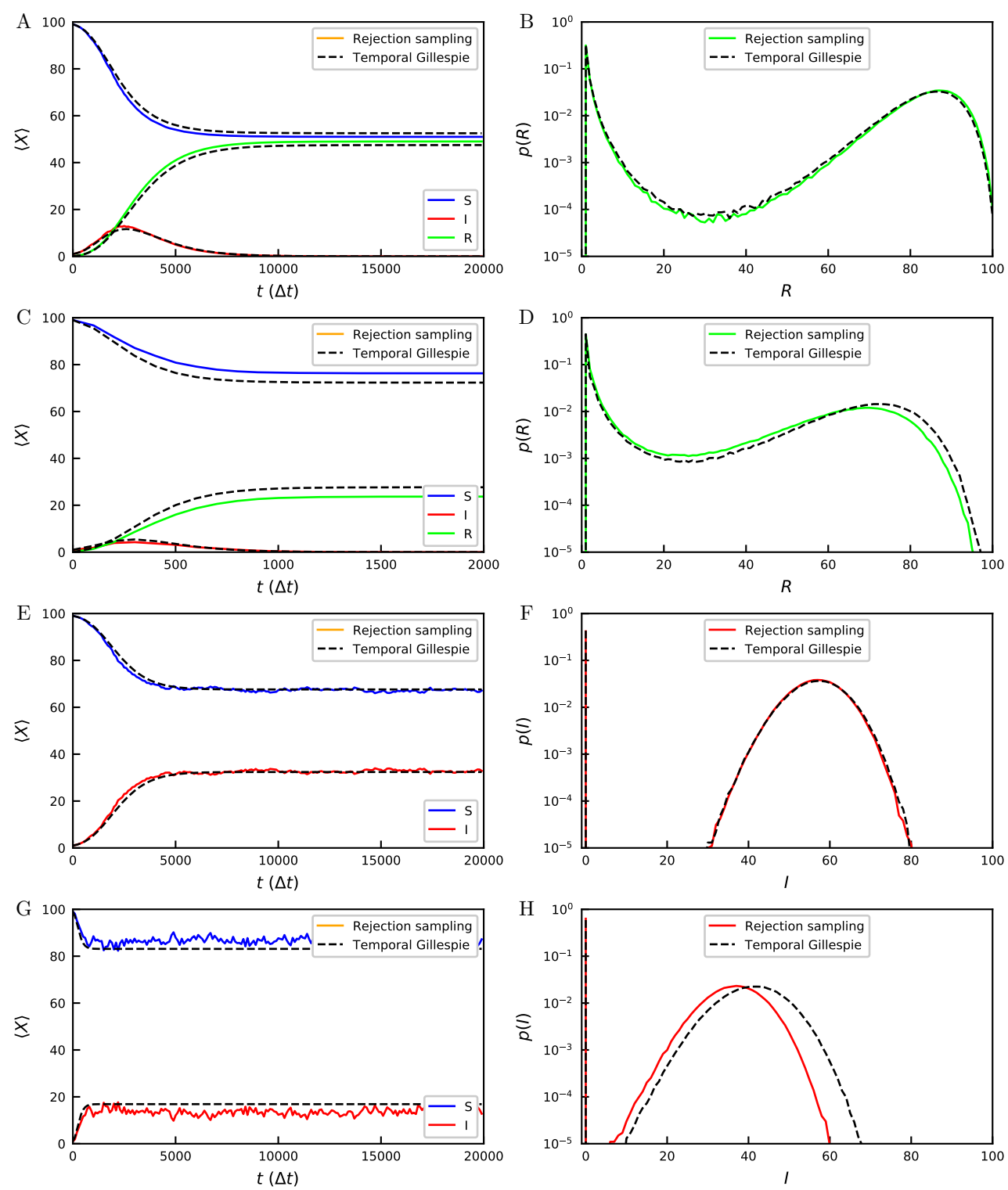

Supplement: S2 Fig — (A)–(D) for a SIR process and (E)–(H) a SIS process. (A),(B),(E), and (F) for βΔt = 10−1 and μΔt = 10−3; (C),(D),(G), and (H) for βΔt = 1 and μΔt = 10−2. (A),(C) Mean number of nodes in each state of the SIR model as function of time. (B),(D) Distribution of final epidemic size (number of recovered nodes when I = 0) in the SIR model. (E),(G) Mean number of nodes in each state of the SIS model as function of time. (F),(H) Distribution of the number of infected nodes in the stationary state (t →∞) of the SIS model. All simulations were performed 1 000 000 times with the root node chosen at random on an activity driven network consisting of N = 100 nodes, with activities ai = ηzi, where η = 0.1 and zi ~ zi-3.2 for zi ϵ [0.03,1), and a node formed two contacts when active. (PDF) [file pcbi.1007190.s002.pdf]

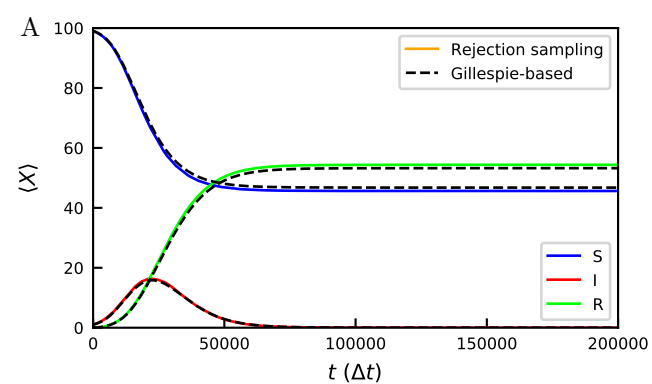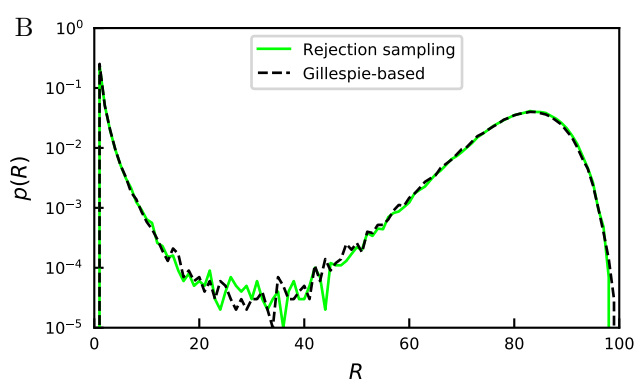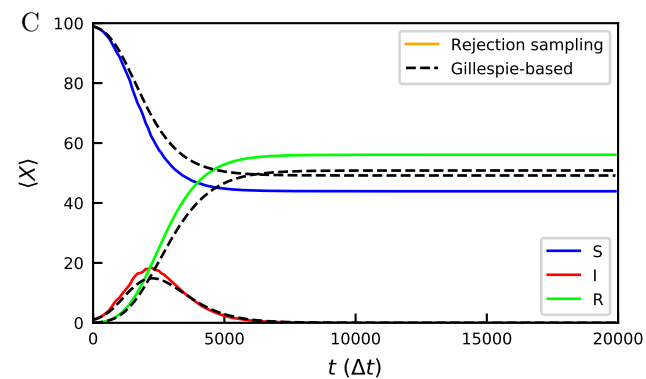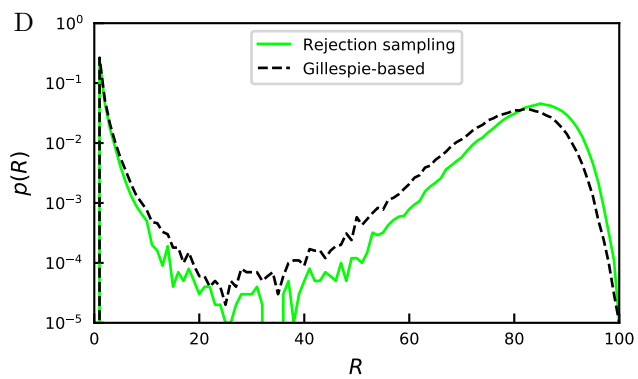

Supplement: S3 Fig — (A),(C) Mean number of nodes in each state as function of time in the SIR model with Weibull distributed recovery times (Sec. 6: “Non-Markovian processes”); the parameter controlling the precision of the temporal Gillespie algorithm was set to ϵ = 0 (quasi-exact). (B),(D) Distribution of final epidemic size (number of recovered nodes when I = 0). (A),(B) βΔt = 10−2 and μΔt = 10−4; (C),(D) βΔt = 10−1 and μΔt = 10−3. The outcome of the rejection sampling algorithm approaches that of the temporal Gillespie algorithm as βΔt and μΔt become smaller. All simulations were performed 100 000 times with the root node chosen at random on an activity driven network consisting of N = 100 nodes, with activities ai = ηzi, where η = 0.1 and zi ~ zi-3.2 for zi ϵ [0.03,1), and a node formed two contacts when active. Nodes’ recovery times followed Eq. (20) with γ = 1.5 and the length of a time-step was Δt = 1 s. (PDF) [file pcbi.1007190.s003.pdf]
